# Sociality to Reach Objects and to Catch Meaning

**DOI:** 10.3389/fpsyg.2019.00838

**Published:** 2019-04-24

**Authors:** Chiara Fini, Anna M. Borghi

**Affiliations:** ^1^Department of Dynamic and Clinical Psychology, Faculty of Medicine and Psychology, Sapienza University of Rome, Rome, Italy; ^2^Institute of Cognitive Sciences and Technologies, National Research Council, Rome, Italy

**Keywords:** WAT theory, abstract concept, body, social tool, words as tools, bodily space, embodied cognition, grounded cognition

## Abstract

Sociality influences both concrete and abstract concepts acquisition and representation, but in different ways. Here we propose that sociality is crucial during the acquisition of abstract concepts but less for concrete concepts, that have a bounded perceptual referent and can be learned more autonomously. For the acquisition of abstract concepts, instead, the human relation would be pivotal in order to master complex meanings. Once acquired, concrete words can act as tools, able to modify our sensorimotor representation of the surrounding environment. Indeed, pronouncing a word the referent of which is distant from us we implicitly assume that, thanks to the contribution of others, the object becomes reachable; this would expand our perception of the near bodily space. Abstract concepts would modify our sensorimotor representation of the space only in the earlier phases of their acquisition, specifically when the child represents an interlocutor as a real, physical *“ready to help actor*” who can help her in forming categories and in explaining the meaning of words that do not possess a concrete referent. Once abstract concepts are acquired, they can work as social tools: the social metacognition mechanism (awareness of our concepts and of our need of the help of others) can evoke the presence of a *“ready to help actor*” in an implicit way, as a predisposition to ask information to fill the knowledge gaps.

## Introduction

Sociality is pivotal for survival and for well-being of our species. It would be difficult to deny that sociality permeates a cognitive process like language, since when talking we need to have an interlocutor, i.e., a person that takes part to the conversation/dialogue with us. In contrast, the role of sociality for processes such as perceiving, categorizing and thinking, is not always sufficiently emphasized. Concepts are “bricks” to build an internal world; they serve to filter the surrounding world, to understand the incoming stimuli for acting and to create new systems of meanings. Here we will argue that sociality is relevant for the formation and representation of concrete and abstract concepts (e.g., “bottle,” “fantasy”), but in different ways. We will first clarify what we intend with concrete and abstract concepts, then we will formulate our theoretical proposal, illustrate evidence supporting it and discuss some open issues.

### Concrete and Abstract Concepts

Concepts have been defined as the “glue” that links our current with our past experience (Murphy, 2002). We intend them as distributed patterns of multimodal experiences, forms of re-enactment of past sensorimotor experiences with their referents (Barsalou, 1999; Borghi, 2005). Concepts play a predictive role (Gallese and Lakoff, 2005): re-enacting our past experiences we can prepare ourselves to interact with a given object or entity. Hence, our concept of computer links our current writing experience with previous ones; in addition, possessing a concept of computer helps us to form expectations and predictions on how to interact with a novel computer.

We will here focus on the distinction between abstract and concrete concepts (e.g., “table” vs. “justice”). We do not intend such a distinction as a dichotomy but we rather conceive it as blurred and not stable, for a number of reasons (Wiemer-Hastings et al., 2001; Barsalou, 2008; Crutch et al., 2013; Borghi et al., 2017). First of all, because concepts are variable and dynamic entities. Second, because each concept can include a mix of concrete and abstract aspects: for example, the concept “dog” can evoke patterns of interaction with the animal, but also more abstract feelings related to possessing a pet etc. Finally, studies performed in our and in other labs have recently shown that the dimension of abstractness and concreteness are typically highly correlated and difficult to disentangle from other dimensions, and that different kinds of abstract concepts exist (Ghio et al., 2013, 2018; Mellem et al., 2016; Borghi et al., 2018c; Desai et al., 2018; Villani et al., 2019; Villani et al., unpublished). Nevertheless, some concepts can be defined as mostly abstract, others as mostly concrete.

On the negative side, abstract concepts are typically less associated than concrete ones to sensorial and perceptual modalities (Barsalou, 2003; Connell and Lynott, 2012), they are typically less imageable (Paivio, 1990) and evoke less Body-Object interactions (BOI) (Siakaluk et al., 2008). On the positive side, abstract concepts are more complex and refer to relations rather than to single, perceptually bounded referents (Borghi and Binkofski, 2014), abstract words are generally acquired later (AoA) and more through linguistic explanations than through perception, i.e., indicating their physical referents (MoA) (Wauters et al., 2003; Della Rosa et al., 2010). Finally, abstract concepts evoke more than concrete ones “social metacognition” (Borghi et al., 2018b,c), i.e., the metacognitive feeling that our knowledge is not adequate (Shea, 2018) and that we need others – possibly authoritative others – to complement it (Prinz, 2012). The preparation to ask information to others seems to be expressed in the activation of the mouth effector. A number of studies, from our lab and other labs, provide evidence that abstract concepts processing involves the activation of the mouth motor system (Borghi et al., 2011; Ghio et al., 2013; Granito et al., 2015; Borghi and Zarcone, 2016; Barca et al., 2017; Mazzuca et al., 2018; see for a review Borghi et al., 2018a). For example, in a recent study, Zannino et al. (unpublished) have shown that articulatory suppression slows down processing of abstract but not of concrete concepts, confirming the importance of inner speech for abstract language processing. In addition to the social metacognition mechanism, other mechanisms might underlie abstract concepts processing: the social experience of word acquisition might be re-enacted, leading to a re-activation of the mouth motor system. Alternatively, the complexity of abstract words might require to re-explain their meaning to ourselves, through the mediation of inner speech.

In sum: in our view both concrete and abstract concepts are grounded in perception-action, in sociality and in linguistic experience, even though the weight of the sensorimotor experience is higher for concrete concepts, that for the social and linguistic experience higher for abstract concepts. In this paper we will focus on the role social experience plays in acquisition and representation of both kinds of concepts.

## The Proposal: Words as Social Tools and Sociality

The main thesis of this paper, that we will articulate and defend, is that acquisition, learning, and representation of both concrete and abstract concepts rely and are influenced by social experience. However, we will qualify this social experience and contend that different kinds of social relationships are involved during processing of concrete and abstract concepts.

Let us consider concrete concepts first. In our view the social dimension is less important for the acquisition of concrete concepts compared to that of abstract ones, since the referents of concrete concepts are perceptually similar and are clearly bounded objects. There is a clear and unique relation between the concept and the referent, that can be autonomously learned. For example, children can form the category of “entities that move on their own,” even if learning the correspondent word, e.g., “animals,” can contribute to refine and render more compact their category (Mirolli and Parisi, 2011). Hence, the linguistic and social input is obviously pivotal in order to learn concrete words, while it is important but not as crucial as it is for abstract concepts in order to form pre-linguistic categories. Once concrete concepts and words have been acquired, their implicit reference to sociality is so strong that it can influence and modify the representation of our bodily space. Indeed, once we have acquired concrete concepts, we can use corresponding words to implicitly ask others to collaborate. Concrete words can thus be used similarly to tools. For example, instead of reaching a far object with a physical instrument we can reach it thanks to a word: pronouncing a concrete word we might induce others to give us objects that we cannot reach. Hence the implicit reference of concrete words to the social dimension can modulate and change the perception of our bodily space, extending it. The impact of using concrete words on shaping our representation of the environment is in our view much more relevant than that of abstract words.

Taking into account abstract concepts (e.g., “fantasy,” “freedom”), we argue, instead, that sociality is crucial for their acquisition. Since the referents of abstract concepts are not perceptually similar and are not clearly bounded objects, we need the others’ linguistic and social input in order to form categories. Consistently, we will advance the new hypothesis that abstract concepts might include a sensorimotor representation that affects the perception of the environment. Because in the early phases of abstract concepts acquisition we might need the another person sufficiently close to us to explain the word meaning, this might impact our space representation. Indeed, we would implicitly assume the “real” presence of a social referent, at least in the earlier phases of conceptual acquisition. The presence of another person, his/her explanations, would be fundamental in order to allow us to form concepts composed by a variety of heterogeneous events and situations, as the members of abstract categories are. Once abstract concepts are acquired, we contend that they always refer to the social dimension, but in a more implicit way. Differently than for concrete words, we might not be able to use abstract words as physical instruments, e.g., to ask others for an object. However, at a metacognitive level we might be less satisfied of our knowledge related to abstract than to concrete concepts, and we might want the help of authoritative others to fill these gaps. Hence, we may continue to need others to complement the gaps of our knowledge (Table 1).

**Table 1:** This table illustrates the social components of concrete and abstract concepts during and after their acquisition.

	Concrete Concept	Abstract Concept
Acquisition	Sociality−	Sociality +
Post-acquisition	The other as concrete instrumental referent	The other as intellectual referent
	Tools to re-arrange the space	Social tools to re-arrange our social relationships

One important note: in distinguishing between concrete and abstract concepts we mentioned “their referents.” Because we intend words as tools that modify our relationship with the surrounding world, it is worth of note that we do not intend referents as something static, that is simply out there in the world. Words are not only pointers to referents, they are rather tools that modify the environment and the space. In this perspective, in keeping with Weber and Varela (2002) and Di Paolo (2005), word meaning depends on the specific mode of coupling that each system realizes with its environment, hence on the specific relation between each language-user and the surrounding context. In the next sections we will sketch how conceptual acquisition might occur, highlighting the differences between acquisition of concrete and of abstract concepts, and how sociality is differently involved once abstract and concrete concepts and words have been acquired.

### Developmental Course: From the “Instrumental Interaction” to the “Intellectual Interaction”

In the human being, the social self emerges quite early compared with the other mammals. At 3–6 months infants are already involved in complex interactions with the mother/caregiver (Kaye, 1982). Imitation, turn-taking games, shared attention, anxiety for the separation and use of the adult’s emotional expression to interpret ambiguous events are examples of sophisticated social expressions (Scaife and Bruner, 1975; Walden and Ogan, 1988; Morales et al., 1998). Infants express their needs and desires through the gaze, the sounds and the gestures; these primitive instruments are called by Vygotsky (1978) “psychological tools.” We can also refer to the “psychological tools” as pragmatic capabilities that would represent the precursor of the language acquisition. The early pragmatic achievements involve three type of communication: (1) negotiating an activity (requesting help, an object, or directing another action), (2) taking part to social routines (saying bye-bye), and (3) regulating mutual attention (vocalizing to attract the other’s attention). Tomasello and Call (1997) introduced the above mentioned distinction. With negotiating an activity they meant to depict the scenario of a kid who, in order to reach for an object, vocalizes or looks at the caregiver, because she knows that the last one is the instrument to obtain the target. Taking part to a social routine is more complex compared with negotiating an activity, because the kid has to respect turn taking, to respond adequately to the other and to be part of a shared context, but still there is no “mentalization” of the other. Only when the kid starts to be interested in capturing the other’s attention, she recognizes the other as an individual able to validate common meanings.

Negotiation of activity is the less complex level among the pragmatic abilities, indeed it belongs also to non-human primates since it is based on the instrumental use of language (Tomasello and Call, 1997). At this level, in humans, the mother would be perceived as an acting body with the capacity to optimize the world features serving the infant’s requests. The mother’s body would be the physical bridge with the world. Such a phase is followed by the acquisition of the social participation capability, that still requires a less sophisticated level of intersubjectivity compared with the regulation of mutual attention (Ninio and Snow, 1999). This last ability emerges around one-year of age, when a sort of Copernican Revolution occurs in infants: The object/action, from fully capturing the infant’s attentional focus becomes the instrument to catch the mother’s attention. The mother/caregiver does not resemble anymore a *bodily tool* or a sort of *instrumental referent* to reach the object when it is outside the infant’s reaching area. She rather starts to be considered also as an *intellectual referent*, somebody to draw knowledge from, who can help to build meanings, to acquire the vocabulary and to construe the concepts useful to interpret the daily life. This shift of the infant’s interest opens to the connection between two minds with the beginning of the cultural development.

This is the phase in which language acquisition occurs. Smith et al. (2011) have beautifully illustrated this process in their studies on word learning with a head-mounted eye tracker (Smith et al., 2011; Yu and Smith, 2013) able to capture children’s perspective and point of view. One-year old infants solve the problem of referential ambiguity (many objects in a scene to which the new word could refer) by focusing their attention on single objects; word learning occurs at best when naming events occur during the moments in which one single object is in their view. Furthermore, they learn new words coordinating their looking behavior with their parents looking together at the objects held by themselves or by the others. Hence, word learning is an embodied and social process, in which statistical learning of words is combined with dynamics of attention, and it is characterized by the presence of the other together with that of the word referent.

### Differences in the Acquisition of Concrete and Abstract Concepts?

When the child acquires new concepts and new words, both concrete and abstract, he/she needs the presence of others. Which are, then, the differences in the acquisition of concrete and abstract concepts and words?

We contend that the presence of others is more crucial during the acquisition of abstract concepts, because their members are quite diverse and heterogeneous. Consider the difference between the concepts “table” and “freedom.” Different tables share many similarities, and often they can be reconducted to a prototypical image; so the child can quite easily learn on her own to abstract from the more idiosyncratic features and to form the category of “table.” This does not mean that children learn concepts on their own, solely on the basis of the perceptual inputs. The linguistic and social input is clearly determinant to refine and render more compact the categories they have formed (Mirolli and Parisi, 2006, 2011; Lupyan and Thompson-Schill, 2012), as well as to associate the label “table” with its referent. Even if important, however, the linguistic and social input it is not indispensable in order to form concrete concepts as it is for abstract concepts.

One further difference is that learning of concrete concepts and words typically occurs in presence of an object/entity, the conceptual referent. Abstract words like “freedom,” instead, do not refer to an object with which the child can interact and that the adults/others can indicate. In order to learn concrete concepts a single label might be a sufficient input, while to learn abstract concepts more extended explanations of the word meaning are generally required in order to gather the multiple experiences abstract concepts assemble together. The guidance of the other/adult and of a rich linguistic input is therefore of paramount importance (for more details on this, Pexman et al., 2002; Recchia and Jones, 2012; Borghi and Binkofski, 2014; Borghi et al., 2018c). Recent findings of Bergelson and Swingley (2013) on 6–16 month-old infants are consistent with this idea. They showed videos to children and parents; parents named events in the video and they verified whether infants followed with their gaze the mentioned object. Results showed that parents tended to mention concrete words in presence of their referent; this occurred less frequently for abstract words. Furthermore, while infants seemed to comprehend concrete words already at 6 months (Bergelson and Swingley, 2012), very simple abstract words (e.g., all gone, more) were not learned before 10 months, and there was a sharp increase of learning abilities around 14 months. This increased acquisition ability can be connected to the development of important social competences, such as the capability to follow the gaze of others at around 10 months (Brooks and Meltzoff, 2005; Beier and Spelke, 2012), and with the development of mature forms of joint attention (Carpenter and Call, 2013). Later, the period in which children learn the majority of abstract words, from 3 years onward, is characterized by their increased capability to discriminate reliable sources: they learn to choose competent others to ask information, as literature on testimony clearly shows (Borghi et al., 2018c).

Even if we focused on conceptual acquisition in children, we do not intend to argue that the involvement of sociality during concepts acquisition is limited to young age. Adults also rely on others to learn new concepts, particularly when concepts are more difficult and more abstract. Compared to young children, adults might have better strategies in identifying competent others, and might be more able to benefit of multiple sources – beyond the interaction with others, they can recur to written sources such as books, Internet, repositories such as Wikipedia etc.

Once both concrete and abstract concepts are acquired, they are obviously updated in light of new experiences and information. For example, experiencing new chairs can lead us to restructure our previous concept of “chair”; the same updating mechanism characterizes both concrete and abstract concepts, even if these last remain more variable, not only between individuals but also for the same individual. The main difference is that concrete words are linked to specific and clearly bounded referents, and because of this once we have learned words we plausibly need others only to communicate with, not to further understand/renegotiate the word meaning.

### Acquisition of Abstract Concepts and Representation of the Space

We have seen that for the acquisition of abstract concepts the presence of others is fundamental. Now the question is whether this can have an effect on the representation of the surrounding space. When the infant starts to learn new words and to explore the correspondence among the words and the reality, in order to master a new ability e.g., talking, she requires to have feedback provided by other people. Specifically, abstract concepts, i.e., the “units” of thinking, would be learned by asking meanings to an *intellectual referent*, usually the mother or a caregiver. Here the social dimension is particularly crucial, because the kid needs another person to acquire meanings and to frame these meanings inside categories in order to interpret the reality. The need of an *intellectual referent* in the acquisition of abstract concepts might induce the child to have an internal physical representation of a “*ready to help actor*” and such representation might weaken when the kid learns to master her question marks and thinking becomes a private act.

We propose that the peculiar modality of acquisition of abstract concepts and words might affect children’s sensorimotor representation of the environment. Indeed, the thinking ability develops in a real human relation, between actors in flesh and bones. The idea is that abstract concept could shape the space perception when the child moves the first steps toward their acquisition, in other words when the physical presence of the *intellectual referent* is crucial. In this phase, when hearing an abstract word, the child would automatically represent/ask for the *“ready to help actor”* endowed with intellectual but also instrumental abilities. The bodily/instrumental potential of the *“ready to help actor”* might determine a re-configuration of the physical reality. In older children and adults, the automatic “instrumental” representation of *“ready to help actor*” would be less strong and the social component in the language acquisition would remain detectable in the sub-threshold mouth motor activation. Understanding abstract concepts would include a more internalized strategy and consequently the process would be a more private experience.

For these reasons it can be hypothesized that when a young kid pronounces or listens an abstract concept and immediately after she is asked to express a sensorimotor judgment, i.e., how much Near/Far is an object, this object would be perceptually filtered by the kid’s body and also by the “ready to help actor’s body.” Later, such sensorimotor co-representation will be less pronounced, and listening/pronouncing abstract concepts would affect the sensorimotor representation to a lesser degree and depending on the abstract concepts meanings. For example, the words “freedom/oppression” might expand/shrink a physical space, or enlarge/reduce a hole, not because the kid imagines a real interlocutor endowed with bodily and knowledge resources, but because of the influence of semantic meaning on the perceptual processes.

## The Word as Form of Action

Both concrete and abstract concepts are grounded in the perception-action system (Caligiore et al., 2010), but for concrete concepts the sensorimotor component is more important than for abstract ones, which are more detached from sensorial modalities (Barsalou, 2003). A study by Connell and Lynott (2012) provides evidence of this different relevance of sensorimotor experience. They collected norms asking participants to determine to what extent they experienced words through each of the five senses. They demonstrated that the so-called concreteness effect, i.e., the advantage of concrete over abstract words, depends on perceptual strength; abstract words are typically less associated to the sensory modalities compared to concrete ones. This does not exclude that the sensorimotor component is important also for abstract concepts. This it is very clear if we consider abstract concepts such as “near/far,” “some,” “more,” but it is true also for concepts like “freedom,” that might re-enact sensorimotor experiences such as running, crossing a border, breaking chains etc.

It is known that the motor system is activated when producing and reading words and that this activation can even be specific to different word types (Pulvermüller et al., 2001; Hauk and Pulvermüller, 2004; Hauk et al., 2004; Shtyrov et al., 2004; see for reviews Barsalou, 2008; Fischer and Zwaan, 2008; Toni et al., 2008; Meteyard et al., 2012; Barsalou, 2016). Specifically, hearing a word seems to be associated with activation of its articulatory motor program, and understanding an action word seems to lead to the immediate and automatic thought of the action to which it refers (Pulvermüller, 2005). A word can vehicle a meaning mapped in a somatotopic manner: it is the case of action words, e.g., “to kick” vs. “to lick.” Alternatively, words can have as referent an affordable object “a cup,” still evoking a motor interaction that recruits a specific effector. Such sensorimotor component in the language is permeated of interpersonal motor resonance, meaning that the words, like the bodies, can scale our representation of the environment by taking into account our own and the other’s action potential. Evidence like this indicates that words are grounded in action (Gallese, 2008; Glenberg and Gallese, 2012).

However, this is not the whole story. Words are not only grounded in action, they can be considered also as a form of action themselves (see Borghi et al., 2013, for extensive discussion on this; Clark, 1998; Dove, 2018). With words we can orient and potentiate our thoughts, modify the opinions and attitudes of others and more generally change the state of the world. We propose that both concrete and abstract words can be used as tools.

More specifically, concrete words can be used as physical tools, i.e., to reach for objects. When using a concrete word, the visual representation of the object might just not demand a motor behavior to the self if the object is located outside our own acting area, but it could also trigger the sensorimotor representation of another actor able to act upon it. In this section we will describe how this representation of concrete words, that explains their nature of “tools,” has an effect on space representation. We will also explain how abstract concepts/words can be instead intended as social tools, that do not impact our spatial representation to the same extent as concrete words but that we use to rely and evoke others.

### Concrete Words as Tools and Their Influence on Space Categorization

#### Influence of Physical Tools and of the Presence of Others on Space Categorization

We propose that words can be intended as physical tools, that extend our spatial representation. Since seminal work by Wittgenstein (1961) and Vygotsky (1978), other authors have claimed that words can be considered as kinds of tools (e.g., Clark, 1998; Tylén et al., 2010). The novelty of our point of view, illustrated in previous work, is to claim that this characteristics of words leads to an expansion of the near space (Borghi and Cimatti, 2010; Borghi et al., 2013; Scorolli et al., 2016). Here we will delimit this claim, arguing that an expansion of the near space occurs only for concrete and not for abstract words. To present our argument, it is important to briefly review studies on tool use and space categorization.

The body is our bridge with the world, it allows us to enact goal directed behavior. Another body, able to act like us is processed with intrinsic action potentialities tailored in response to the space context. Evidence pointed out that we represent the body of others as endowed with our same action potentialities: an object may namely afford a suitable motor act not only when it is close to our own hand but also, crucially, to the hand of an avatar or of another person (Coello and Delevoye-Turrell, 2007; Costantini et al., 2011a; Cardellicchio et al., 2012). In the peripersonal space it has been shown that the presence of others is able to modulate our predisposition to act toward a graspable object (Costantini et al., 2011b).

Studies on tool use revealed that the boundary between near and far space is a flexible one, and that using tools to reach for objects leads to an extension of our representation of peripersonal (near) space (Berlucchi and Aglioti, 1997; Berti and Frassinetti, 2000; Maravita and Iriki, 2004; Farnè et al., 2005; see also Arbib et al., 2009). This expansion of the peripersonal space does not occur only when we use tools, but also when we observe others using them. The simple observation of someone reaching an object with a tool, extends our perception of the peripersonal space (Costantini et al., 2011a; Bloesch et al., 2012). Recent evidence indicates that this flexibility of our spatial representation is not confined to the peripersonal, near space, but it is extended to the extrapersonal space. Fini et al. (2014) have shown that seeing a human body, potentially able to cover a distance in the extrapersonal space (outside the reaching space), can reduce our space categorization. These findings indicate that another human body is a relevant stimulus automatically processed as a like-us intentional agent. Since we are social animals, we likely assume the same agent to have a collaborative attitude toward us. In presence of “*another like-me body”* who is located close to us, we perceptually build a spatial layout that takes into account the impact of another person on our goal directed behavior. That is, the other is processed as a “social arm-tool” to pass a cup that we cannot reach, or as “a social-leg tool” to walk to the soccer ball if we are too tired to cover the distance.

#### Concrete Words as Ethereal Tools

If the body is our bridge with the world, the word is our bridge with the others. In general we communicate with other people through verbal language or gestures if we are too much distant to be heard, still we can scream. Either the words and the body serve to communicate meanings, but the words are able to convey more complex meanings and allow us to be more precise, for example to refer to a specific object among many. When an adult talks usually there is at least interlocutor to promote a dialogue.

Let us consider two different roles among the many that language can play. Words can help us to find a solution to reach concrete aims, e.g., “Can you pass me the pen?,” or to create new knowledge e.g., “Do you think that this object/entity/event belongs to this category? What does this word mean?” In the first case, the sentence invokes the help of an *instrumental referent* who can give us the pen, making the pen closer to us and expanding our near space. In the second case, the sentence invokes the help of an *intellectual referent*, who help us to learn the meaning of new words. These two functions can be summarized as follows: (1) The word, like a physical tool or like the body of another agent, serves to reach distant objects that are outside our action domain; (2) The word is useful to understand the meaning of words, to create and build new conceptual networks. We propose that the first function of language concerns primarily concrete concepts, while the second function of words concerns both concrete and abstract concepts, but is particularly prominent for abstract ones.

Empirical evidence supports the idea that concrete words work as physical tools. Experimental results obtained by Scorolli et al. (2016) indicate that an object located on a table in the border space the participant’s reaching area, can be perceived as closer not only when participants are grabbing a rake, or when they can press a button to make the object appear, but crucially also when participants simply pronounce the name of the object. When we pronounce a word, typically “another-like us” listens our speech. Assuming that he/she has a cooperative attitude, he/she becomes an instrument to reach for objects located far away from us. We propose that such social dimension would be automatically activated also when simply pronouncing a word. In other terms, the presence of the other can be implicit, i.e., language can re-evoke the presence of another person, even if there is no physical trace of this. While it has been shown that concrete words can affect our perception of the environment through the intrinsic social dimension that they have, so far there is no evidence on how the social dimension of abstract words can induce a similar effect.

## Abstract Words as Social Tools: The Mechanism of Social Metacognition

So far we have claimed that sociality is more crucial for abstract than for concrete concepts acquisition, and that, once concepts are acquired, we might use concrete words as tools. Concrete words namely implicitly evoke the presence of others who may help us in reaching objects, and this impacts our representation of the reachable space. While the representation of abstract words likely does not impact and modulate the borders of our bodily space, in our view sociality continues to influence abstract concept representation in different ways.

Once abstract concepts have been acquired, to what extent does their processing involve the presence of others? Can this presence be evoked only in an implicit way? Let us consider separately the three mechanisms that we briefly illustrated in section. We propose that these mechanisms, that are not mutually exclusive and can co-exist, underlie abstract concepts processing, and explain why the mouth motor system is activated (for details, Borghi et al., 2018b,c). These mechanisms are: (A) Re-enactment of the linguistic/social acquisition process. Because we would re-enact the past experience of acquiring the concept, it is unlikely that the use of such a mechanism is influenced by the real presence of others when we process concepts. The others are simply evoked re-acting situation in which their presence and contribution facilitated word acquisition. (B) Re-explanation of the meaning of the abstract words, possibly through the use of inner speech. This mechanism does not imply the physical presence of others, since it involves the use of speech for ourselves. (C) Social metacognition. Basically, we would tell to ourselves that our concepts are not adequate, and try to find solutions outside from ourselves. The mouth activation would be due to the motor preparation to ask information to others. It is certainly possible, and needs to be tested with appropriate experiments, that the presence of others is influential when such a social metacognition mechanism is active. The presence of others who might potentially fulfill our needs can render the activation of the mouth motor system more pronounced. However, in purely theoretical terms such a mechanism could work also in absence of real others.

In sum, sociality would be involved in all these mechanisms that we hypothesize to be at the basis of abstract concepts processing. In all cases the involvement of sociality would have a bodily impact, determining a selective activation of the mouth motor system. However, the involvement of sociality differs in extent across the three mechanisms. For the first two mechanisms not only language would become internalized, in Vygotskian terms (Vygotsky, 1986), but also the reference to a possible companion/other. Things differ for the social metacognition mechanism, for which we hypothesize that the presence of real, physical others, although not necessary, can determine a stronger activation of the mouth motor system. In presence of real others, we might namely prepare ourselves to ask them information more promptly than if we implicitly refer to possible others.

## Conclusion

According to Words As social Tools proposal (WAT), concrete concepts like “glass” or “table” have a sensorial well-defined referent and their acquisition stems from the sensorimotor experience of the physical object/entity to which concepts refer. Abstract concepts are more detached from the sensorial experience, and evoke more social and linguistic experience than concrete ones (Borghi and Binkofski, 2014; Borghi et al., 2018b,c). Both the abstract and the concrete concepts are embodied. The embodied counterpart of the abstract concept is manifested in the mouth motor activation, trace of the inner language acquired through the social relation. The embodied counterpart of the concrete concept is manifested in the whole body.

The main thesis of this paper is that sociality influences both concrete and abstract concepts acquisition and representation, but in different ways. We revised developmental literature showing how the point of contact between the infant and the surrounding reality/environment is the mother/caregiver. Far from being considered just an *instrumental referent* to reach an object, she becomes an *intellectual referent* to catch meanings. The role of the other as intellectual referent is particularly crucial for the acquisition of abstract concepts: due to the heterogeneity of their members and to their detachment from sensory modalities they are more difficult to learn relying exclusively on the perceptual inputs.

Once concrete and abstract concepts have been acquired, sociality continues to be determinant for their representation. We briefly illustrated theoretical proposals and evidence showing how concrete words can act as tools (Borghi and Cimatti, 2010; Borghi et al., 2013; Scorolli et al., 2016): similarly to human bodies (Costantini et al., 2011b; Cardellicchio et al., 2012; Fini et al., 2015), they affect the sensorimotor representation of the surrounding environment leading to an extension of the near space.

**FIGURE 1:**
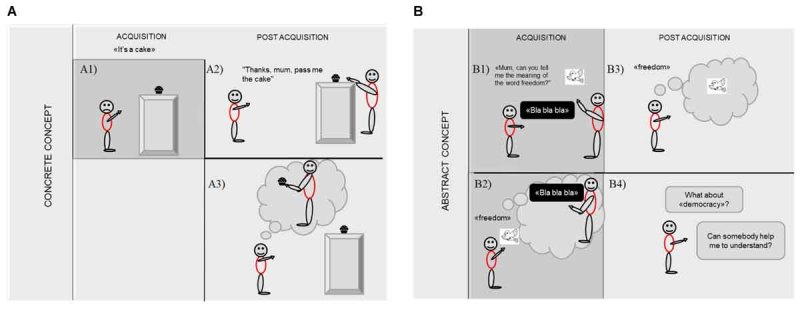
**(A)** The mechanism underlying the acquisition of concrete concepts: **(A1)** The child has the perceptual experience of the concrete referent and acquires the correct word associated with it, **(A2)** The child asks help to the mother as an *instrumental referent*, to reach the concrete object that has been conceptually acquired, and **(A3)** The child associates with the concrete concept the sensorimotor simulation of the interaction of her/other’s body, and this interpersonal motor resonance can re-shape the environmental representation. **(B)** The mechanism underlying the acquisition of abstract concepts: **(B1)** The child asks to the mother to explain the meaning of an abstract word and the mother tries to explain this meaning also by using concrete referents, **(B2)** The child when using and/or listening a new concept and/or observing a specific referent that refers to it, re-enacts the experience of the mother as intellectual referent. In these phase the intellectual referent might be implicitly perceived as real, inducing an interpersonal motor resonance that can re-shape the environmental representation, **(B3)** The child has acquired the conceptual knowledge, she masters the new meaning, and **(B4)** The child when learning new concepts, can ask for the presence of a *“ready to help actor*” in an implicit way, as a predisposition to ask information to fill the knowledge gaps (social metacognition).

Here, we propose that also abstract concepts, like concrete ones, might influence the perception of the environment but following two different modalities. In the earlier phases of abstract concepts acquisition, the child might represent an interlocutor as a real, physical *“ready to help actor*,” with a consequent interpersonal bodily representation of the physical reality (sensorimotor modalities) until the moment in which the dialogue between the infant and the real interlocutor becomes internalized. When the infant masters a solipsistic inner language, three possible mechanisms underlie and explain the activation of the mouth motor system during abstract concepts processing. For the re-enactment and re-explanation mechanisms the reference to a possible companion/other would be implicitly evoked. The social metacognition mechanism can evoke the presence of a *“ready to help actor*” in an implicit way, but it can lead to a stronger activation of the mouth motor system in presence of real others, to whom to prepare to ask information and help (Figure 1).

New research is necessary to investigate how the social component of abstract concept evolves, from being external and more embodied, to be internal and more semantic and how this is reflected in a different perception of the world.

## Author Contributions

All authors listed have made a substantial, direct and intellectual contribution to the work, and approved it for publication.

## Conflict of Interest Statement

The authors declare that the research was conducted in the absence of any commercial or financial relationships that could be construed as a potential conflict of interest. The reviewer LW declared a past collaboration with one of the authors AB.
